# Clustering psychopathology in male anabolic–androgenic steroid users and nonusing weightlifters

**DOI:** 10.1002/brb3.3040

**Published:** 2023-05-07

**Authors:** Marie Lindvik Jørstad, Morgan Scarth, Svenn Torgersen, Harrison Graham Pope, Astrid Bjørnebekk

**Affiliations:** ^1^ Anabolic Androgenic Steroid Research Group, National Advisory Unit on SUD Treatment, Division of Mental Health and Addiction Oslo University Hospital Oslo Norway; ^2^ Anabolic Androgenic Steroid Research Group, Section for Clinical Addiction Research, Division of Mental Health and Addiction Oslo University Hospital Oslo Norway; ^3^ Department of Psychology University of Oslo Oslo Norway; ^4^ Biological Psychiatry Laboratory McLean Hospital Belmont Massachusetts USA; ^5^ Harvard Medical School, Department of Psychiatry Boston Massachusetts USA

**Keywords:** anabolic steroids, doping, hierarchical clustering, personality pathology, psychopathology

## Abstract

**Introduction:**

Prior research has demonstrated that personality disorders and clinical psychiatric syndromes are common among users of anabolic–androgenic steroids (AAS). However, the prevalence, expression, and severity of psychopathology differ among AAS users and remain poorly understood. In this study, we examine the existence of potential clinically coherent psychopathology subgroups, using cluster procedures.

**Methods:**

A sample of 118 male AAS users and 97 weightlifting nonusers was assessed using the Millon Clinical Multiaxial Inventory‐III (MCMI‐III), measuring personality disorders and clinical syndromes. Group differences in MCMI‐III scales were assessed using Wilcoxon‐Mann–Whitney tests and Fisher's exact test. Agglomerative hierarchical clustering was used to identify clusters based on MCMI‐III scale scores from the whole sample.

**Results:**

AAS users displayed significantly higher scores on all personality disorder (except narcissistic) and clinical syndrome scales compared to nonusing weightlifters. The clustering analysis found four separate clusters with different levels and patterns of psychopathology. The “no psychopathology” cluster was most common among nonusing weightlifters, while the three other clusters were more common among AAS users: “severe multipathology,” “low multipathology,” and “mild externalizing.” The “severe multipathology” cluster was found almost exclusively among AAS users. AAS users also displayed the highest scores on drug and alcohol dependence syndromes.

**Conclusions:**

AAS users in our sample demonstrated greater psychopathology than the nonusing weightlifters, with many exhibiting multipathology. This may pose a significant challenge to clinical care for AAS users, particularly as there appears to be significant variation in psychopathology in this population. Individual psychiatric profiles should be taken into consideration when providing treatment to this group.

**Significant Outcomes:**

- As a group, AAS users displayed markedly greater psychopathology than nonusing weightlifters.
- Multipathology was common among AAS users.
- Four different subgroups of personality profiles were identified with distinct patterns of pathology and severity.

**Limitations:**

- The cross‐sectional nature of the study precludes inferences about causality.
- The study is limited by possible selection bias, as participants choosing to be involved in research may not be entirely representative for the group as a whole.
- The study is vulnerable to information bias, as the results are based on self‐report measures and interviews.

## Graphical abstract

In a study comparing long‐term AAS users and nonusers weightlifters on the Millon Clinical Multiaxial Inventory, we found markedly greater psychopathology among AAS users than among weightlifting nonusing. The cluster analysis revealed four different subgroups of personality profiles with distinct patterns of personality pathology and severity.

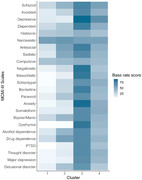

## 1 INTRODUCTION

Anabolic–androgenic steroids (AAS) are a family of drugs that includes the male hormone testosterone, together with hundreds of synthetic analogues of testosterone (Kicman, 2008; Pope, Wood, et al., 2014). AAS are best known because they are used by professional and recreational athletes for their muscle‐building effects (Bhasin et al., 1996). Prevalence estimates suggest that around 3–6% of young men in most Western countries have used AAS (Kanayama et al., 2010; Pope, Kanayama, et al., 2014; Pope, Wood, et al., 2014; Sagoe et al., 2014), typically in supraphysiologic doses, thus, increasing the risk of potential side‐effects (Pope, Wood, et al., 2014). These range from acne, sleep disturbances, and gynecomastia (Ip et al., 2011; Parkinson & Evans, 2006) to more serious complications such as cardiovascular effects (Baggish et al., 2017; Melsom et al., 2022; Thiblin et al., 2015), prolonged hypogonadism (Kanayama et al., 2015; Rasmussen et al., 2016), poorer cognitive performance (Bjørnebekk et al., 2019; Kanayama et al., 2013), and brain structural and functional abnormalities (Bjørnebekk et al., 2021, 2017; Pope et al., 2021; Scarth & Bjørnebekk, 2021; Westlye et al., 2017).

In addition, many AAS users suffer from a range of psychiatric disorders. Studies suggest a higher prevalence of personality psychopathology such as antisocial, histrionic, and borderline personality disorder in male and female (Choi & Pope, 1994; Cooper et al., 1996; Hallgren et al., 2015; Hauger et al., 2021; Perry et al., 2003; Piacentino et al., 2022; Scarth, Jørstad, et al., 2022; Yates et al., 1990) AAS users compared to nonusers. In addition, various studies have reported an elevated prevalence of anxiety, paranoia, depression, irritability, aggression, hostility, violence, and body image disturbances in AAS users (Chegeni et al., 2021; Choi et al., 1990; Choi & Pope, 1994; Cooper et al., 1996; Gestsdottir et al., 2021; Griffiths et al., 2018; Malone et al., 1995; Moss et al., 1992; Murray et al., 2016; Pagonis et al., 2006; Perry et al., 1990, 2003; Pope et al., 2021; Pope & Katz, 1988, 1994). A minority of users are also reported to develop mania and/or hypomania, occasionally associated with psychotic symptoms, during AAS use (Malone et al., 1995; Pope & Katz, 1988, 1994), and major depression, occasionally associated with suicidal ideation, most often occuring during AAS withdrawal (Borjesson et al., 2020; Gestsdottir et al., 2021; Patel et al., 2021; Petersson et al., 2006; Pope & Katz, 1988, 1994; Thiblin et al., 1999). However, most studies on the associations between AAS use and psychopathology find that the majority of AAS users show minimal psychiatric effects, while a minority display more severe psychopathology (Pope, Wood, et al., 2014). In addition, most studies have looked at single psychiatric diagnoses instead of also focusing on comorbidity, which may not fully capture the complex and overlapping nature of psychopathology among users of AAS. It is now well recognized that psychiatric diagnoses tend to occur co‐morbidly, and that looking at dimensions or subtypes of psychopathology might be a better alternative than looking at individual diagnoses alone (Caspi et al., 2014). Thus, to better capture potential associations between AAS use and psychopathology, we sought to look for possible psychopathological profiles based on response patterns, as has previously been performed in patients with other types of substance‐use disorders using cluster analyses (McMahon, 2008).

The purpose of the current study was to investigate personality pathology and specific psychiatric syndromes in a group of male AAS users and a comparison group of weightlifters reporting no use of AAS, using the MCMI‐III. In particular, we sought to identify potential clinically coherent psychopathology subgroups within the dataset, using cluster procedures. We hypothesized that (1) AAS users as a group would display more severe psychopathology than weightlifting controls, and (2) cluster analysis would reveal different categories of psychopathology within the AAS using group. In particular, we hypothesized that we would find one cluster characterized by multipathology, one cluster with low or no psychopathology, and distinct clusters with elevated scores on either externalizing or internalizing traits.

## 2 MATERIALS AND METHODS

### 2.1 Participants

A group of 118 current or past male AAS users and a comparison group of 97 nonusing male weightlifters completed the Millon Clinical Multiaxial Inventory‐III (MCMI‐III) as a part of a longitudinal study investigating brain, medical, and psychiatric effects associated with long‐term use of AAS at Oslo University Hospital. The sample is partly overlapping with the one described in Bjørnebekk et al. (2021). The data collection was performed at two different time points from 2013 to 2015 and from 2017 to 2019. At both time points, the participants were recruited through posters and flyers distributed at selected gyms in Oslo, and through online forums and websites addressing heavy resistance training and/or AAS use. Prior to enrollment, all participants received an information brochure with a complete description of the study, and written informed consent was collected from all subjects. The participants were compensated with 1000 Norwegian kroner at time point 1 and with 500 Norwegian kroner at time point 2. AAS use or nonuse was assessed among the entire sample from which our group of participants was drawn. Testing for AAS drug use was conducted at the WADA‐accredited laboratory at Oslo University Hospital as described in detail previously (Bjørnebekk et al., 2021). The criteria used to determine external androgen use were: (1) urine samples positive for synthetic testosterone compounds, and (2) a testosterone to epitestosterone ratio (T/E) greater than 15. In previous publications (Bjørnebekk et al., 2021, 2023), our group found excellent agreement between these laboratory results and participants’ self‐report, with no controls testing positive for AAS. One AAS user was excluded based on the MCMI‐III's validity exclusion criteria (Jankowski, 2002), leaving 117 AAS users and 97 nonusers for analysis. Seven of the AAS users were unable to come to Oslo for testing, and thus, performed only the assessment inventories. Consequently, information regarding the nature of their AAS use and training regimens is missing.

### 2.2 Instruments

**The Millon Clinical Multiaxial Inventory‐III (MCMI‐III)** is a 175‐item, true–false, self‐reported inventory for measuring personality disorders and identifying existing syndromes (Millon, 2006). The 175 items comprise a validity scale, three scales to detect response bias, 14 personality disorder scales, and 10 clinical syndrome scales, grouped by severity. These scales closely parallel the classification of the main Axis I and all Axis II disorders in the American Psychiatric Association's Diagnostic and Statistical Manual of Mental Disorders, 4th edition (American Psychiatric Association, 1994). Each scale is scored using base rate (BR) scores, measuring the prevalence and severity of a psychological characteristic on a continuum, and is representative of the actual prevalence of the particular attribute in the psychiatric population. The scales range from BR score 1 to 115, where a score of 60 is the median score obtained by psychiatric patients (Jankowski, 2002). A score **greater than or equal to** 75 on a scale indicates the presence of a trait or syndrome (Millon, 2006). The disclosure index scale (scale x), one of the scales to detect response bias, measures the participant's response style, and is based on raw scores. Scores less than 34 on the disclosure index scale indicate excessive secretiveness, whereas scores greater than 178 on the same scale indicate that the participant is too self‐revealing, and when MCMI‐III is used in clinical samples, such scores would invalidate the test results (Jankowski, 2002). Seven AAS users and 17 nonusing weightlifters scored higher than 34 on the disclosure index scale, while two AAS users scored lower than 178. However, as this was an exploratory study on a non‐clinical sample, these participants were not excluded from our analyses.

We also administered a **semistructured interview,** specifically designed for this study, covering demographic data, weight training history, health‐related information, and history of prescription drug use. Also, the history and characteristics of AAS use were assessed with questions assessing age at onset of use, weekly AAS dose estimates (mg), years of use, and whether the AAS use was current or previous.

### 2.3 Statistical analyses

Group differences in demographic data were evaluated with independent‐sample *t‐*tests on continuous variables and chi‐square tests for independence on categorical data using SPSS Statistics 26. The remaining statistical analyses were performed using R version 4.0.3 (R Core Team, 2018). Comparisons between AAS users and nonusers on MCMI‐III scales were assessed using Wilcoxon–Mann–Whitney tests to account for the non‐normal distributions on these measures. Scores were dichotomized using the clinical cut‐off of BR greater than or equal to 75 and compared between groups using Fisher's exact test to account for the small number of expected elevated scores, particularly in the group of nonusing weightlifters. *p*‐Values were adjusted using the Benjamini–Hochberg procedure to control for the false discovery rate. The same analyses were repeated to compare previous and current AAS users.

Agglomerative hierarchical clustering with Euclidean distances and Ward's D2 method was used to identify clusters based on BR scores on all MCMI‐III scales from the whole sample. The optimal number of clusters was determined using a combination of the NbClust package (Charrad et al., 2014), which calculates 25 indices of optimal cluster number, in addition to a within‐cluster sum of squares scree plot. Chi‐square tests were used to compare the prevalence of cluster membership between AAS users and nonusing weightlifters, and effect sizes were calculated using the phi coefficient.

## 3 RESULTS

### 3.1 Sample characteristics

The groups did not significantly differ in age, but the nonusing weigtlifters had on average about 2 years more education than the AAS users (Table 1). Although the nonuser group reported significantly more weight training hours per week, AAS users were significantly stronger on both strength measures, which is in line with the anabolic properties of AAS. Prescription drug use was significantly more common among the AAS users, where 38% reported such use.

**TABLE 1:** Demographics, strength training, prescription drug use and AAS use

	Nonusing weightlifters (n = 97)		AAS users (n = 117)			95% Confidence interval		
Sample characteristics	Mean	SD	Mean	SD	*t*	LL	UL	*p*
Demographics								
Age, years	35.1	9.9	36.5	10.1	−1.05	−4.2	1.3	.294
Education, years^a^	16.5	2.6	14.6	2.9	5.05	1.2	2.7	.000
Height, cm^b^	181.0	6.7	181.3	6.9	−0.37	−2.2	1.5	.714
Weight, kg^b^	91.0	11.7	97.8	15.2	−3.59	−10.6	−3.1	.000
Body mass index^b^	27.8	3.4	29.7	4.1	−3.68	−3.0	−0.9	.000
Strength training, min/week^a^	418.2	230.1	335.9	186.0	2.84	25.2	139.6	.005
Bench press 1 RM^c^	137.6	25.1	168.4	33.8	−7.32	−39.0	−22.5	.000
Squats 1 RM^d^	177.5	37.2	213.2	53.2	−5.27	−49.1	−22.4	.000
	**n(%)**		**n(%)**		***X* ^2^**			***p***
Prescription drug use^g^	7 (7.2)		41 (38.0)		25.3			.000
Weekly AAS dose estimate, mg^h^								
<300 (low)			11 (11.0)					
300–1000 (medium)			52 (52.0)					
>1000 (high)			37 (37.0)					
			**Mean**	**Median**	**Range**			
Age at onset of AAS use^e^			22.6	20.0	12–55			
Years of AAS use^f^			10.6	8.0	.6–35			

AAS, anabolic androgenic steroids; LL, lower limit; RM, repetition maximum; UL, upper limit. ^a‐h^Data availability for the different measures varied. Mean values are based on the following number of non‐using weightlifters/AAS users: 97/109^a^, 97/112^b^, 95/104^c^, 89/93^d^, 113^e^, 111^f^, 97/108^g^, 100^h^

### 3.2 Characteristics of AAS use

In the AAS user group, the mean age of onset of AAS use was 22.6 years (Table 1). The mean cumulative lifetime duration of AAS use was 10.6 years. Using criteria proposed by Pope and Katz (1994), we categorized the users weekly doses into “low” (<300 mg of testosterone equivalent per week), “medium” (300–1000 mg/week), and “high” (>1000 mg/week). Among the 100 users for whom these data were available, we classified 11% as using low, 52% as using medium, and 37% as using high weekly doses.

### 3.3 MCMI‐III‐scales

The AAS group displayed significantly higher scores than the nonuser group on all MCMI‐III‐scales except for the narcissistic personality disorder scale (Table 2). The antisocial, aggressive, passive‐aggressive, self‐defeating, avoidant, depressive, and schizotypal personality disorder traits, in that order according to the *z*‐values, were the scales on which the AAS users differed most from nonusers, based on the median score. Among the clinical syndrome scales, the drug and alcohol dependency scales differed most strongly between AAS users and nonusers. Similar findings emerged when assessing the number of individuals scoring above the clinical cut‐off of BR greater than or equal to 75 on the various scales (Supporting Information Table S1).

**TABLE 2:** MCMI‐III base rate medians, interquartile range, and results of the Wilcoxon–Mann–Whitney tests

	Nonusing weightlifters (n = 97)		AAS Users (n = 117)		*Z*	*p*
MCMI‐III scales	Median	IQR	Median	IQR		
Moderate personality disorder scales						
Schizoid	36.0	[12.0, 60.0]	60.0	[24.0, 72.0]	−4.372	.00
Avoidant	12.0	[0.0, 36.0]	36.0	[12.0, 74.0]	−4.618	.00
Depressive	0.0	[0.0, 40.0]	40.0	[0.0, 78.0]	−4.430	.00
Dependent	30.0	[10.0, 50.0]	40.0	[20.0, 65.0]	−2.790	.01
Histrionic	38.0	[30.0, 42.0]	40.0	[34.0, 44.0]	−2.153	.03
Narcissistic	57.0	[51.0, 61.0]	57.0	[53.0, 61.0]	−0.732	.46
Antisocial	22.0	[8.0, 38.0]	60.0	[30.0, 73.0]	−7.832	.00
Aggressive (Sadistic)	17.0	[9.0, 34.0]	60.0	[26.0, 63.0]	−6.609	.00
Compulsive	41.0	[34.0, 46.0]	44.0	[36.0, 49.0]	−2.249	.03
Passive aggressive (Negativistic)	15.0	[0.0, 22.0]	30.0	[15.0, 60.0]	−5.479	.00
Self‐defeating (Masochistic)	0.0	[0.0, 20.0]	20.0	[0.0, 68.0]	−4.855	.00
Severe personality pathology scales						
Schizotypal	0.0	[0.0, 40.0]	40.0	[0.0, 63.0]	−5.435	.00
Borderline	10.0	[0.0, 30.0]	30.0	[10.0, 60.0]	−5.743	.00
Paranoid	24.0	[0.0, 36.0]	48.0	[12.0, 64.0]	−4.712	.00
Moderate clinical syndrome scales						
Anxiety	0.0	[0.0, 20.0]	40.0	[0.0, 80.0]	−4.596	.00
Somatoform	0.0	[0.0, 30.0]	60.0	[0.0, 64.0]	−4.762	.00
Bipolar: Manic	24.0	[12.0, 48.0]	60.0	[24.0, 64.0]	−4.632	.00
Dysthymia	0.0	[0.0, 20.0]	20.0	[0.0, 64.0]	−4.621	.00
Alcohol dependence	15.0	[0.0, 30.0]	45.0	[30.0, 65.0]	−7.347	.00
Drug dependence	15.0	[15.0, 30.0]	60.0	[30.0, 67.0]	−7.996	.00
Post‐traumatic stress disorder	0.0	[0.0, 15.0]	15.0	[0.0, 63.0]	−4.465	.00
Severe syndrome scales						
Thought disorder	0.0	[0.0, 30.0]	30.0	[0.0, 63.0]	−4.051	.00
Major depression	0.0	[0.0, 20.0]	20.0	[0.0, 61.0]	−4.793	.00
Delusional disorder	25.0	[0.0, 25.0]	25.0	[0.0, 63.0]	−3.179	.00

AAS, anabolic androgenic steroids; IQR, interquartile range.

*p*‐Values were adjusted using Benjamini–Hochberg procedure.

### 3.4 MCMI‐III clusters

**FIGURE 1:**
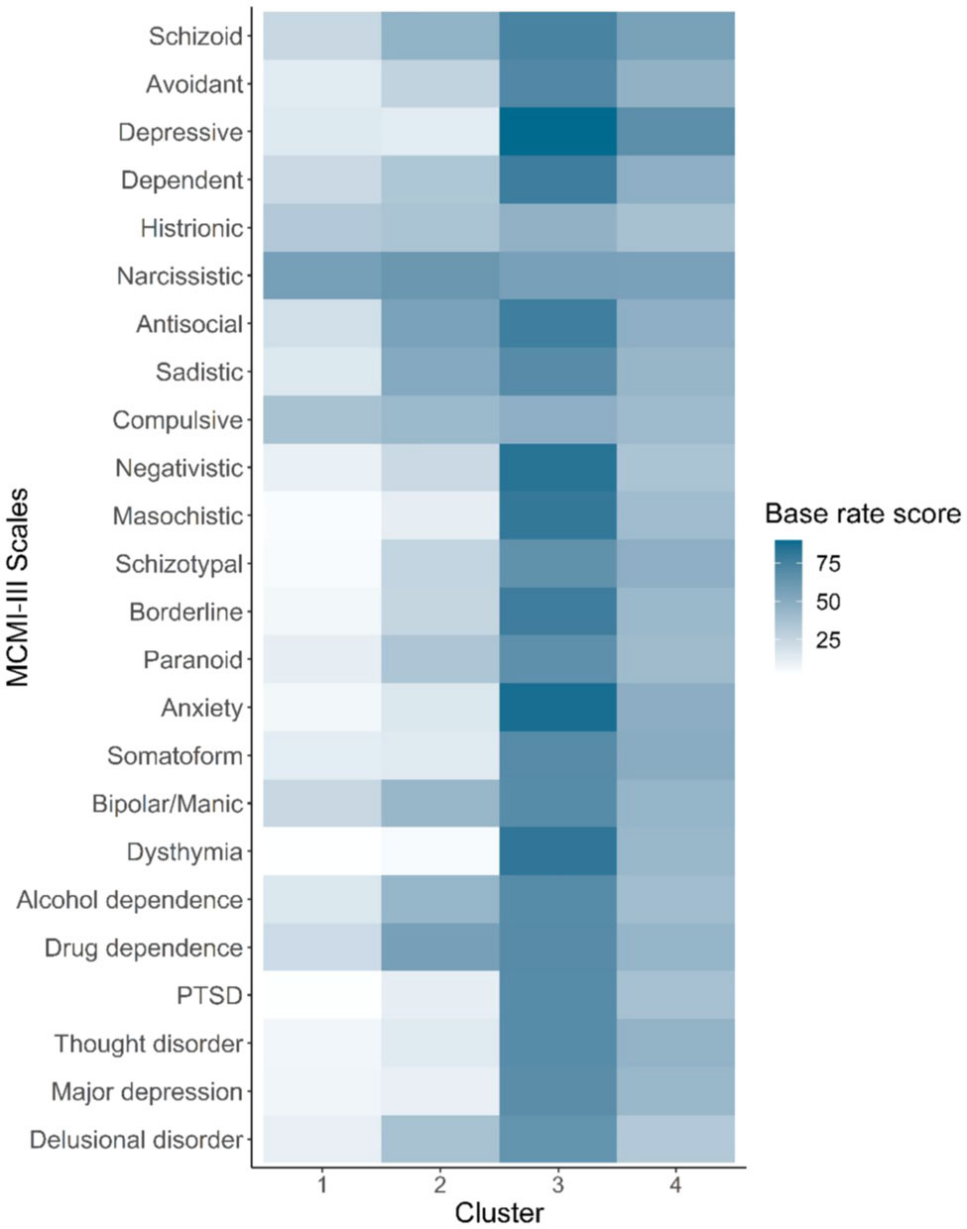
Cluster analysis heat map. Dark blue means a higher score on the scales. PTSD, post‐traumatic stress disorder.

When performing the cluster analysis, we found that eight indices from the NbClust package suggested that two clusters were optimal, while seven indices suggested that four clusters were optimal (Charrad et al., 2014) (Supporting Information Figure S1). The within‐cluster sum of squares scree plot indicated that four clusters yielded significantly less within‐cluster variation than the two‐cluster solution (Supporting Information Figure S2). The decision to select four clusters was also retrospectively supported by the distinctive and clinically relevant characteristics of the clusters (Supporting Information Figure S3). The results of the agglomerative hierarchical clustering are presented in Table 3 and Figure 1, with an overview of groups of participants in each cluster presented in Table 4.

**TABLE 3:** Cluster analysis, mean base rate scores, and number of scales above the clinical cut‐off (≥75) within each cluster

	Cluster 1 (n = 89) No psychopathology		Cluster 2 (n = 46) Mild externalizing		Cluster 3 (n = 23) Severe multipathology		Cluster 4 (n = 56) Mild multipathology	
MCMI‐III scales	Mean (SD)	BR ≥ 75 n (%)	Mean (SD)	BR ≥ 75 n (%)	Mean (SD)	BR ≥ 75 n (%)	Mean (SD)	BR ≥ 75 n (%)
Moderate personality disorder scales								
Schizoid	24.4 (20.6)	1 (1.1)	46.2 (23.5)	5 (10.9)	74.1 (16.0)	13 (56.5)	56.3 (23.0)	10 (17.9)
Avoidant	14.3 (13.2)	0 (0.0)	27.0 (25.5)	2 (4.3)	71.4 (25.2)	13 (56.5)	46.2 (25.5)	14 (25.0)
Depressive	15.1 (22.4)	0 (0.0)	13.5 (18.9)	0 (0.0)	90.0 (21.3)	22 (95.7)	67.6 (17.6)	22 (39.3)
Dependent	23.5 (17.1)	1 (1.1)	34.2 (21.1)	1 (2.2)	77.6 (16.5)	16 (69.6)	48.2 (23.0)	11 (19.6)
Histrionic	33.1 (9.7)	0 (0.0)	36.1 (10.9)	0 (0.0)	46.3 (6.3)	0 (0.0)	37.6 (9.6)	0 (0.0)
Narcissistic	57.4 (8.2)	5 (5.6)	62.1 (10.2)	5 (10.9)	56.6 (21.0)	4 (17.4)	56.1 (15.3)	6 (10.7)
Antisocial	20.5 (13.6)	0 (0.0)	55.6 (19.0)	6 (13.0)	77.3 (15.5)	14 (60.9)	48.1 (25.5)	10 (17.9)
Aggressive (Sadistic)	15.6 (14.0)	0 (0.0)	51.5 (13.5)	1 (2.2)	69.5 (9.7)	8 (34.8)	44.0 (21.3)	0 (0.0)
Compulsive	37.2 (10.2)	0 (0.0)	42.0 (8.8)	0 (0.0)	48.0 (6.1)	0 (0.0)	41.6 (9.5)	0 (0.0)
Passive aggressive (Negativistic)	10.5 (12.5)	0 (0.0)	23.6 (14.5)	0 (0.0)	84.0 (14.1)	20 (87.0)	35.8 (22.4)	4 (7.1)
Masochistic	3.9 (11.7)	0 (0.0)	12.2 (18.6)	0 (0.0)	80.9 (10.8)	21 (91.3)	40.4 (29.8)	11 (19.6)
Severe personality pathology scales								
Schizotypal	5.0 (14.1)	0 (0.0)	26.2 (24.3)	0 (0.0)	65.7 (14.9)	8 (34.8)	47.8 (23.1)	0 (0.0)
Bordeline	7.4 (10.7)	0 (0.0)	25.2 (17.4)	0 (0.0)	77.5 (17.7)	16 (69.6)	42.5 (20.0)	0 (0.0)
Paranoid	11.9 (16.7)	0 (0.0)	35.6 (24.0)	0 (0.0)	66.7 (7.6)	2 (8.7)	41.4 (26.2)	2 (3.6)
Moderate clinical syndrome scales								
Anxiety	7.2 (16.3)	2 (2.2)	16.3 (26.7)	5 (10.9)	87.0 (14.3)	22 (95.7)	48.7 (30.3)	19 (33.9)
Somatoform	13.0 (23.1)	0 (0.0)	14.6 (24.8)	0 (0.0)	69.6 (10.0)	4 (17.4)	49.3 (22.5)	0 (0.0)
Bipolar: Manic	24.2 (21.2)	0 (0.0)	43.7 (22.0)	0 (0.0)	69.1 (12.1)	6 (26.1)	44.9 (23.0)	1 (1.8)
Dysthymia	2.0 (6.8)	0 (0.0)	4.8 (12.8)	0 (0.0)	82.0 (13.1)	18 (78.3)	42.9 (26.8)	8 (14.3)
Alcohol dependence	16.4 (17.9)	0 (0.0)	44.1 (21.4)	3 (6.5)	69.2 (15.9)	11 (47.8)	40.2 (25.0)	6 (10.7)
Drug dependence	22.6 (15.7)	0 (0.0)	57.0 (16.8)	7 (15.2)	69.4 (12.6)	8 (34.8)	44.7 (24.9)	4 (7.1)
Post‐traumatic stress disorder	3.0 (9.7)	0 (0.0)	11.8 (21.9)	0 (0.0)	68.9 (13.8)	7 (30.4)	37.4 (25.1)	2 (3.6)
Severe syndrome scales								
Thought disorder	7.6 (14.1)	0 (0.0)	14.7 (17.4)	0 (0.0)	68.9 (10.1)	5 (21.7)	45.7 (20.7)	0 (0.0)
Major depression	8.3 (17.9)	0 (0.0)	11.3 (18.8)	0 (0.0)	67.9 (16.5)	5 (21.7)	42.8 (23.8)	1 (1.8)
Delusional disorder	11.1 (17.5)	0 (0.0)	37.1 (25.4)	0 (0.0)	64.4 (5.1)	1 (4.3)	33.0 (27.5)	0 (0.0)

BR, base rate.

**TABLE 4:** Participants in each cluster

	Nonusing weightlifters n = 97	AAS Users n = 117			
	n (%)	n (%)	χ2	*φ*	*P*
Cluster 1 (n = 89) «No psychopathology»	63 (64.9)	26 (22.2)	38.11	0.43	<.001
Cluster 2 (n = 46) «Mild externalizing»	13 (13.4)	33 (28.2)	6.04	0.18	.014
Cluster 3 (n = 23) «Severe multipathology»	2 (2.1)	21 (17.9)	12.35	0.26	<.001
Cluster 4 (n = 56) «Mild multipathology»	19 (19.6)	37 (31.6)	3.38	0.05	.07

AAS, anabolic androgenic steroids

The first cluster (no psychopathology) was characterized by low scores on all scales except for the narcissistic personality pattern. The second cluster (mild externalizing) was characterized by moderately high scores on the narcissistic, antisocial, and sadistic personality pattern scales, together with the drug dependence syndrome scale. The third cluster (severe multipathology) was characterized by scores in the clinical range on most of the scales. Particularly, high scores were seen on the depressive, negativistic, masochistic, antisocial, and dependent personality pattern scales, the borderline severe personality scale, and the anxiety and dysthymic syndrome scales. Between 60.9 and 95.7% of the individuals in this cluster scored above BR 75 on the aforementioned scales. The participants in this cluster also showed the highest mean score on the drug dependence and alcohol dependence syndrome scales. The fourth cluster (mild multipathology) was characterized by a high mean score on the depressive personality pattern scale, and moderately high scores on the schizoid, narcissistic, dependent, and antisocial personality pattern scales, as well as the somatoform and anxiety syndrome scales. About two thirds of the nonusing weightlifters, but relatively few AAS users, fell into the “no psychopathology” cluster. “Severe multipathology” was seen almost exclusively in the AAS users.

### 3.5 Characteristics of MCMI‐III personality subgroups

An analysis comparing the characteristics of the participants in the previously described clusters (Supporting Information Table S2) found that participants in cluster 1 (no psychopathology) had significantly more education than the remaining groups. Additionally, participants in cluster 3 (severe multipathology) differed significantly in age at onset of AAS use as compared to participants in cluster 1 (no psychopathology) and 2 (mild externalizing), with a mean age at onset of AAS use of 18.18 in the former group. Apart from this difference, no other statistically significant differences were found between the groups with regard to age, total years used, or weekly dose estimate.

### 3.6 Current and previous AAS use

We possessed data on 104 of the 117 AAS users with regard to current versus previous AAS use. A total of 72 individuals reported that they were current users, and 32 reported only previous use. When comparing these two subgroups on the various MCMI‐III measures, we found no significant differences on median scores (Supporting Information Table S3) or on the proportion of individuals exhibiting elevated scores (BR ≥ 75) (Supporting Information Table S4) on any measure.

## 4 DISCUSSION

The findings from the present study using the MCMI‐III demonstrated marked differences in psychopathology between the groups of 117 AAS‐using and 97 nonusing weightlifters. AAS users showed significantly higher BR scores on all personality and syndrome scales, except for the narcissistic scale, with a much higher number of scale scores reaching the clinical cut‐off (BR ≥ 75). The cluster analysis revealed four different subgroups of psychopathology profiles with distinct patterns of personality and syndrome scale scores. A comparison between individuals reporting current AAS use with those reporting previous AAS use showed no significant differences between these groups. This suggests that the MCMI‐III was detecting stable traits in the AAS‐using population, rather than state‐dependent effects associated with current AAS use.

Nearly one‐third of the AAS sample, as opposed to about one‐tenth of the weightlifting controls, exhibited elevated scores on the depressive personality and/or anxiety syndrome scales, with the highest scores in the two multipathology clusters. This finding is consistent with previous studies documenting anxiety and mood disorders among AAS users (Amaral et al., 2022; Gestsdottir et al., 2021; Ip et al., 2011; Pope & Katz, 1994; Scarth, Jørstad et al., 2022). In particular, it is likely that hypogonadism precipitated by AAS withdrawal may cause depressive syndromes with attendant anxiety in some men (Kanayama et al., 2015; Malone et al., 1995; Pope & Katz, 1988; Rasmussen et al., 2016). Of note, a 30‐year follow‐up study of former elite male athletes in power sports suggests possible long‐term effects of AAS use on mental health, in that former AAS users more often sought professional help for mental problems, such as depression and anxiety, compared to athletes with no history of AAS exposure (Lindqvist et al., 2013). Also of note, it has been reported that among AAS users who sought help to quit AAS use, most did so as a result of mental health problems, with depression and anxiety being the most prominent effects described (Havnes et al., 2019). It has also been reported that AAS users are more prone to suicide or suicidal ideation than nonusers (Gestsdottir et al., 2021; Patel et al., 2021; Petersson et al., 2006; Thiblin et al., 1999), and that this risk increases if personality disorders are present (Borjesson et al., 2020).

In our study, the most pronounced difference in personality pathology between AAS users and nonusers was the antisocial personality pattern. The findings of high scores on antisocial personality disorder have been presented on a part of this sample previously (Hauger et al., 2021), where our research group found that antisocial personality traits could be an important mediator in the relationship between AAS use, aggression, and violence. In addition to antisocial personality disorder, sadistic, and borderline personality disorder were scales that differed markedly between AAS users and controls. These findings are consistent with previous studies of male AAS users (Borjesson et al., 2020; Cooper et al., 1996; Hallgren et al., 2015; Perry et al., 2003; Yates et al., 1990), and one study of female users (Scarth, Havnes et al., 2022). Similar profiles have been found in individuals with substance‐use disorders (Verheul, 2001), suggesting an overlap in personality factors between AAS users and users of other illicit substances.

A striking finding of our study was a large number of AAS users exhibiting multiple areas of psychopathology. About half of the AAS users had psychopathology profiles fitting into two multipathology clusters. This observation appears congruent with the description of a “general psychopathology” *p*‐factor as described by Caspi et al. (2014), and the fact that severe disturbances tend to be comorbid (Caspi et al., 2014; Rosenstrom et al., 2019). Accumulating evidence suggests that the combination of externalizing and internalizing pathology poses a greater risk of life impairment, subsequent psychiatric disorders, criminal offenses, and other self‐reported problems (Caspi et al., 2014; Sourander et al., 2007). These findings accord well with the results of the present study, where individuals in the severe multipathology cluster showed not only high scores on all scales, but also high‐risk behaviors such as high drug dependence scores and low age at onset of AAS use.

Polypharmacy is common among AAS users (DiClemente, 2014; Dodge & Hoagland, 2011; DuRant et al., 1995; Pope et al., 2012; Pope & Katz, 1994; Sagoe et al., 2015; Skarberg et al., 2009), as was also the case in our sample, where the median BR score on “drug dependence” was 60 for the AAS users. The majority of AAS users scored below the cut‐off, but 14.4% displayed elevated scores, with the most problematic substance use seen in the severe multipathology cluster. This finding resembles the findings of another recent study where ongoing use of narcotic agents and alcohol was more common in AAS users diagnosed with a personality disorder (Borjesson et al., 2020). It has also been reported that patients with substance‐use disorders who also exhibited AAS dependence showed more severe personality pathology than substance‐use disorder patients not using AAS (Scarth, Havnes et al., 2022). In studies of inmates (Havnes et al., 2020) and of patients in treatment for substance‐use disorders (Havnes, Jørstad, et al., 2020), individuals using AAS were found to exhibit more severe overall substance use than AAS nonusers. The direction of causality underlying these associations is complex, likely reflecting a combination of genetic liability, environmental, and drug exposure effects.

Last, as has also been found in other studies (Pope et al., 2014), it is worth noting that 22.2% of the AAS users had a personality profile fitting into the “no psychopathology” cluster, meaning that they had low scores overall, except for scores on the narcissistic personality pathology scale. Narcissism has previously been linked to appearance‐ and fitness‐enhancing behaviors in general (Martinovic et al., 2022; Miller & Mesagno, 2014). The finding of no psychopathology in a group of the AAS users in our sample is expected, as it resembles several other studies finding that the majority of AAS users reported minimal psychiatric effects, or finding few significant psychopathological differences between weightlifting controls and AAS users as a whole (Bahrke et al., 1996; Moss et al., 1992; Tricker et al., 1996; Windfeld‐Mathiasen et al., 2022). The differences that we observed in psychiatric morbidity both between AAS users and weightlifting controls, and within the group of AAS users, may be affected by many different factors linked to AAS use. For instance, AAS users experience more physical health problems compared to controls, which may contribute to increased psychopathology (Barnett et al., 2012; Horwitz et al., 2019). Interestingly, we found no significant differences among AAS users in the four clusters with respect to lifetime duration of AAS use, with even individuals in the “no psychopathology” cluster reporting a mean of more than 10 years of cumulative AAS exposure. A potential confounding variable in such comparisons may be level of education, as this, in turn, is often linked to socioeconomic status, and as lower education level is associated with an increased risk for psychotic and mood disorders (Kivimäki et al., 2020). In our study, the highest level of education was found in the weightlifting control group, and in the “no psychopathology” cluster, both of which had low psychopathology scores.

### 4.1 Limitations

The cross‐sectional nature of the study precludes inferences about causality, and longitudinal studies will be required to better delineate the causal pathways involved in the psychopathology observed. The present study is also limited by possible selection bias, in that the AAS users and nonusers who chose to participate in the study may not have been entirely representative of their respective source populations in the community. The findings are also vulnerable to information bias, in that the results are based on interviews and self‐report measures that could not be confirmed with objective measures. However, given the magnitude of the differences observed among the groups on the various measures, and the relatively large number of participating AAS users, it seems unlikely that the differences observed could be attributable purely to these sources of bias. It is also important to note that the MCMI‐III was designed and standardized for use in clinical populations, but has also proven to be useful in research settings (Millon, 2006). Nevertheless, our findings of reported elevated scales in this research population should not be considered as diagnoses, but rather as general indicators of pathology.

## 5 CONCLUSIONS

In a study comparing long‐term AAS users and nonusing weightlifters on the Millon Clinical Multiaxial Inventory, we found markedly greater psychopathology among AAS users than among weightlifting nonusers. The cluster analysis revealed four different subgroups of personality profiles with distinct patterns of personality pathology and severity. These findings may help to inform health care providers about potential personality profiles of AAS users, to guide interventions for individual users. In particular, health care providers should be aware that a proportion of AAS users show complex personality pathology, characterized by severe internalizing and externalizing pathology and consequently a substantial need for mental health care services.

### CONFLICT OF INTEREST STATEMENT

All authors declare that they have no conflict of interest.

### PEER REVIEW

The peer review history for this article is available at https://publons.com/publon/10.1002/brb3.3040

## Supporting information


**Supplementary Figure 1**: NbClust showing the optimal number of clusters.
**Supplementary Figure 2**: Elbow plot showing the optimal number of clusters.
**Supplementary Figure 3**. Dendrogram showing how the four clusters divide from each other.
**Supplementary Table 1**. Frequency and percent of MCMI‐III scales above the clinical cut‐off (≥75).
**Supplementary Table 2**: Characteristics of MCMI‐III psychopathology sub‐groups.
**Supplementary Table 3**: Current and previous AAS users, MCMI‐III base rate medians, interquartile range and results of the Wilcoxon‐Mann‐Whitney tests.
**Supplementary Table 4**: Current and previous AAS users, frequency and percent of MCMI‐III scales above the clinical cut‐off (≥75).

## Data Availability

The data that support the findings of this study are not publicly available due to their sensitive nature, where our ethical approval prevents us from sharing data beyond named collaborators. However, upon reasonable request, we will allow necessary insight into the material. Further inquiries can be directed to the corresponding author.

## References

[brb33040-bib-0001] Amaral, J. X. , Deslandes, A. C. , Padilha, M. C. , Vieira Neto, L. , Osorio, L. E. , Aquino Neto, F. R. , & Cruz, M. S. (2022). No association between psychiatric symptoms and doses of anabolic steroids in a cohort of male and female bodybuilders. Drug Testing and Analysis, 14(6), 1079–1088. 10.1002/dta.3230 35092181 PMC9303351

[brb33040-bib-0002] American Psychiatric Association . (1994). Diagnostic and statistical manual of mental disorders (4th ed.). American Psychiatric Association.

[brb33040-bib-0003] Baggish, A. L. , Weiner, R. B. , Kanayama, G. , Hudson, J. I. , Lu, M. T. , Hoffmann, U. , & Pope, H. G. (2017). Cardiovascular toxicity of illicit anabolic‐androgenic steroid use. Circulation, 135(21), 1991–2002. 10.1161/CIRCULATIONAHA.116.026945 28533317 PMC5614517

[brb33040-bib-0004] Bahrke, M. S. , Yesalis, C. E. , & Wright, J. E. (1996). Psychological and behavioural effects of endogenous testosterone and anabolic‐androgenic steroids. An update. Sports Medicine (Auckland, N.Z.), 22(6), 367–390. 10.2165/00007256-199622060-00005 8969015

[brb33040-bib-0005] Barnett, K. , Mercer, S. W. , Norbury, M. , Watt, G. , Wyke, S. , & Guthrie, B. (2012). Epidemiology of multimorbidity and implications for health care, research, and medical education: a cross‐sectional study. Lancet, 380(9836), 37–43. 10.1016/S0140-6736(12)60240-2 22579043

[brb33040-bib-0006] Bhasin, S. , Storer, T. W. , Berman, N. , Callegari, C. , Clevenger, B. , Phillips, J. , Bunnell, T. J. , Tricker, R. , Shirazi, A. , & Casaburi, R. (1996). The effects of supraphysiologic doses of testosterone on muscle size and strength in normal men. New England Journal of Medicine, 335(1), 1–7. 10.1056/NEJM199607043350101 8637535

[brb33040-bib-0007] Bjørnebekk, A. , Kaufmann, T. , Hauger, L. E. , Klonteig, S. , Hullstein, I. R. , & Westlye, L. T. (2021). Long‐term anabolic‐androgenic steroid use is associated with deviant brain aging. Biol Psychiatry Cogn Neurosci Neuroimaging, 6(5), 579–589. 10.1016/j.bpsc.2021.01.001 33811018

[brb33040-bib-0008] Bjørnebekk, A. , Scarth, M. , Neupane, S. P. , Westlye, L. T. , Hullstein, I. R. , Thorsby, P. M. , & Halvorsen, B. (2023). Use of high‐dose androgens is associated with reduced brain‐derived neurotrophic factor in male weightlifters. Neuroendocrinology, 113(1), 36–47. 10.1159/000526418 35944495

[brb33040-bib-0009] Bjørnebekk, A. , Walhovd, K. B. , Jørstad, M L. , Due‐Tønnessen, P. , Hullstein, I. R. , & Fjell, A. M. (2017). Structural brain imaging of long‐term anabolic‐androgenic steroid users and nonusing weightlifters. Biological Psychiatry, 82(4), 294–302. 10.1016/j.biopsych.2016.06.017 27616036

[brb33040-bib-0010] Bjørnebekk, A. , Westlye, L. T. , Walhovd, K B. , Jørstad, M. L. , Sundseth, Ø. Ø. , & Fjell, A. M. (2019). Cognitive performance and structural brain correlates in long‐term anabolic‐androgenic steroid exposed and nonexposed weightlifters. Neuropsychology, 33(4), 547–559. 10.1037/neu0000537 31033318

[brb33040-bib-0011] Börjesson, A. , Möller, C. , Hagelin, A. , Vicente, V. , Rane, A. , Lehtihet, M. , Dahl, M. L. , Gårevik, N. , & Ekström, L. (2020). Male anabolic androgenic steroid users with personality disorders report more aggressive feelings, suicidal thoughts, and criminality. Medicina (Kaunas, Lithuania), 56(6), 265. 10.3390/medicina56060265 32481676 PMC7353874

[brb33040-bib-0012] Caspi, A. , Houts, R. M. , Belsky, D. W. , Goldman‐Mellor, S. J. , Harrington, H. , Israel, S. , Meier, M. H. , Ramrakha, S. , Shalev, I. , Poulton, R. , & Moffitt, T. E. (2014). The *p* factor: one general psychopathology factor in the structure of psychiatric disorders? Clinical Psychology Science, 2(2), 119–137. 10.1177/2167702613497473 PMC4209412 25360393

[brb33040-bib-0013] Charrad, M. , Ghazzali, N. , Boiteau, V. , & Niknafs, A. (2014). Nbclust: An R package for determining the relevant number of clusters in a data set. Journal of Statistical Software, 61(6), 1–36. <Go to ISI>://WOS:000349840900001.

[brb33040-bib-0014] Chegeni, R. , Notelaers, G. , Pallesen, S. , & Sagoe, D. (2021). Aggression and psychological distress in male and female anabolic‐androgenic steroid users: A multigroup latent class analysis. Frontiers in Psychiatry, 12, 629428. 10.3389/fpsyt.2021.629428 34149470 PMC8211877

[brb33040-bib-0015] Choi, P. Y. L. , Parrott, A. C. , & Cowan, D. (1990). High‐dose anabolic steroids in strength athletes: Effects upon hostility and aggression. Human Psychopharmacology: Clinical and Experimental, 5(4), 349–356. 10.1002/hup.470050407

[brb33040-bib-0016] Choi, P. , & Pope, H. (1994). Violence toward women and illicit androgenic‐anabolic steroid use. Annals of Clinical Psychiatry, 6(1), 21–25. 10.3109/10401239409148835 7951641

[brb33040-bib-0017] Cooper, C. J. , Noakes, T. D. , Dunne, T. , Lambert, M. I. , & Rochford, K. (1996). A high prevalence of abnormal personality traits in chronic users of anabolic‐androgenic steroids. British Journal of Sports Medicine, 30(3), 246–250. 10.1136/bjsm.30.3.246 8889121 PMC1332342

[brb33040-bib-0018] Diclemente, J. R. (2014). Steroid use, health risk behaviors and adverse health indicators among U.S. high school students. Family Medicine & Medical Science Research, 03(3). 10.4172/2327-4972.1000127

[brb33040-bib-0019] Dodge, T. , & Hoagland, M. F. (2011). The use of anabolic androgenic steroids and polypharmacy. Drug and Alcohol Dependence, 114(2‐3), 100–109. 10.1016/j.drugalcdep.2010.11.011 21232881 PMC3062678

[brb33040-bib-0020] Durant, R. H. , Escobedo, L. G. , & Heath, G. W. (1995). Anabolic‐steroid use, strength training, and multiple drug use among adolescents in the United States. Pediatrics, 96(1 Pt 1), 23–28. https://www.ncbi.nlm.nih.gov/pubmed/7596717 7596717

[brb33040-bib-0021] Gestsdottir, S. , Kristjansdottir, H. , Sigurdsson, H. , & Sigfusdottir, I. D. (2021). Prevalence, mental health and substance use of anabolic steroid users: a population‐based study on young individuals. Scandinavian Journal of Public Health, 49(5), 555–562. 10.1177/1403494820973096 33280527

[brb33040-bib-0022] Griffiths, S. , Jacka, B. , Degenhardt, L. , Murray, S. B. , & Larance, B. (2018). Physical appearance concerns are uniquely associated with the severity of steroid dependence and depression in anabolic‐androgenic steroid users. Drug and Alcohol Review, 37(5), 664–670. 10.1111/dar.12688 29484740

[brb33040-bib-0023] Hallgren, M. , Pope Jr, H G. , Kanayama, G. , Hudson, J. I. , Lundin, A. , & Källmén, H. (2015). Anti‐social behaviors associated with anabolic‐androgenic steroid use among male adolescents. European Addiction Research, 21(6), 321–326. 10.1159/000433580 26113433

[brb33040-bib-0024] Hauger, L. E. , Havnes, I. A. , Jørstad, M. L. , & Bjørnebekk, A. (2021). Anabolic androgenic steroids, antisocial personality traits, aggression and violence. Drug and Alcohol Dependence, 221, 108604. 10.1016/j.drugalcdep.2021.108604 33621808

[brb33040-bib-0025] Havnes, I. A. , Bukten, A. , Rognli, E. B. , & Muller, A. E. (2020). Use of anabolic‐androgenic steroids and other substances prior to and during imprisonment: Results from the Norwegian Offender Mental Health and Addiction (NorMA) study. Drug and Alcohol Dependence, 217, 108255. 10.1016/j.drugalcdep.2020.108255 32949884

[brb33040-bib-0026] Havnes, I. A. , Jørstad, M. L. , Mcveigh, J. , Van Hout, M. C. , & Bjørnebekk, A. (2020). The anabolic androgenic steroid treatment gap: A national study of substance use disorder treatment. Subst Abuse, 14, 117822182090415. 10.1177/1178221820904150 PMC7031794 32127749

[brb33040-bib-0027] Havnes, I. A. , Jørstad, M. L. , & Wisløff, C. (2019). Anabolic‐androgenic steroid users receiving health‐related information: Health problems, motivations to quit and treatment desires. Substance Abuse Treatment Prevention, and Policy, 14(1), 20. 10.1186/s13011-019-0206-5 31096999 PMC6524231

[brb33040-bib-0028] Horwitz, H. , Andersen, J. T. , & Dalhoff, K. P. (2019). Health consequences of androgenic anabolic steroid use. Journal of Internal Medicine, 285(3), 333–340. 10.1111/joim.12850 30460728

[brb33040-bib-0029] Ip, E. J. , Barnett, M. J. , Tenerowicz, M. J. , & Perry, P. J. (2011). The Anabolic 500 survey: Characteristics of male users versus nonusers of anabolic‐androgenic steroids for strength training. Pharmacotherapy, 31(8), 757–766. 10.1592/phco.31.8.757 21923602

[brb33040-bib-0030] Jankowski, D. (2002). A beginner's guide to the MCMI‐III. American Psychological Association. 10.1037/10446-000

[brb33040-bib-0031] Kanayama, G. , Hudson, J. I. , Deluca, J. , Isaacs, S. , Baggish, A. , Weiner, R. , Bhasin, S. , & Pope, H. G. (2015). Prolonged hypogonadism in males following withdrawal from anabolic‐androgenic steroids: An under‐recognized problem. Addiction, 110(5), 823–831. 10.1111/add.12850 25598171 PMC4398624

[brb33040-bib-0032] Kanayama, G. , Hudson, J. I. , & Pope, H. G. Jr. (2010). Illicit anabolic‐androgenic steroid use. Hormones and Behavior, 58(1), 111–121. 10.1016/j.yhbeh.2009.09.006 19769977 PMC2883629

[brb33040-bib-0033] Kanayama, G. , Kean, J. , Hudson, J. I. , & Pope, H. G. (2013). Cognitive deficits in long‐term anabolic‐androgenic steroid users. Drug and Alcohol Dependence, 130(1‐3), 208–214. 10.1016/j.drugalcdep.2012.11.008 23253252 PMC3608708

[brb33040-bib-0034] Kicman, A. T. (2008). Pharmacology of anabolic steroids. British Journal of Pharmacology, 154(3), 502–521. 10.1038/bjp.2008.165 18500378 PMC2439524

[brb33040-bib-0035] Kivimäki, M. , Batty, G. D. , Pentti, J. , Shipley, M. J. , Sipilä, P. N. , Nyberg, S. T. , Suominen, S. B. , Oksanen, T. , Stenholm, S. , Virtanen, M. , Marmot, M. G. , Singh‐Manoux, A. , Brunner, E. J. , Lindbohm, J. V. , Ferrie, J. E. , & Vahtera, J. (2020). Association between socioeconomic status and the development of mental and physical health conditions in adulthood: a multi‐cohort study. Lancet Public Health, 5(3), e140–e149. 10.1016/s2468-2667(19)30248-8 32007134

[brb33040-bib-0036] Lindqvist, A. S. , Moberg, T. , Eriksson, B. O. , Ehrnborg, C. , Rosén, T. , & Fahlke, C. (2013). A retrospective 30‐year follow‐up study of former Swedish‐elite male athletes in power sports with a past anabolic androgenic steroids use: a focus on mental health. British Journal of Sports Medicine, 47(15), 965. 10.1136/bjsports-2012-091340 23613517

[brb33040-bib-0037] Malone, D. A. , Dimeff, R. J. , Lombardo, J. A. , & Barry Sample, R. H. (1995). Psychiatric effects and psychoactive substance use in anabolic‐androgenic steroid users. Clinical Journal of Sport Medicine, 5(1), 25–31. http://www.ncbi.nlm.nih.gov/pubmed/7614077 7614077 10.1097/00042752-199501000-00005

[brb33040-bib-0038] Martinovic, D. , Tokic, D. , Martinovic, L. , Rakusic, M. , Kumric, M. , Rusic, D. , Vilovic, M. , Vrdoljak, J. , Ticinovic Kurir, T. , & Bozic, J. (2022). Orthorexia nervosa and its association with narcissism in fitness center users. Eating and Weight Disorders, 27, 2155–2163. 10.1007/s40519-022-01368-9 35103950 PMC8804367

[brb33040-bib-0039] Mcmahon, R. C. (2008). Substance abuse problems, psychiatric symptoms, and post‐treatment status in MCMI psychopathology subgroups of cocaine dependent males. American Journal of Drug and Alcohol Abuse, 34(2), 195–202. 10.1080/00952990701877094 18293236

[brb33040-bib-0040] Melsom, H. S. , Heiestad, C. M. , Eftestøl, E. , Torp, M. K. , Gundersen, K. , Bjørnebekk, A. K. , Thorsby, P. M. , Stensløkken, K. O. , & Hisdal, J. (2022). Reduced arterial elasticity after anabolic‐androgenic steroid use in young adult males and mice. Scientific Reports, 12(1), 9707. 10.1038/s41598-022-14065-5 35690664 PMC9188580

[brb33040-bib-0041] Miller, K. J. , & Mesagno, C. (2014). Personality traits and exercise dependence: Exploring the role of narcissism and perfectionism. International Journal of Sport and Exercise Psychology, 12(4), 368–381. 10.1080/1612197x.2014.932821

[brb33040-bib-0042] Millon, T. (2006). MCMI‐III manual (3rd ed.). NCS Pearson.

[brb33040-bib-0043] Moss, H. B. , Panzak, G. L. , & Tarter, R. E. (1992). Personality, mood, and psychiatric symptoms among anabolic steroid users. American Journal on Addictions, 1(4), 315–324. 10.1111/j.1521-0391.1992.tb00357.x

[brb33040-bib-0044] Murray, S. B. , Griffiths, S. , Mond, J. M. , Kean, J. , & Blashill, A. J. (2016). Anabolic steroid use and body image psychopathology in men: Delineating between appearance‐ versus performance‐driven motivations. Drug and Alcohol Dependence, 165, 198–202. 10.1016/j.drugalcdep.2016.06.008 27364377

[brb33040-bib-0045] Pagonis, T. A. , Angelopoulos, N. V. , Koukoulis, G. N. , & Hadjichristodoulou, C. S. (2006). Psychiatric side effects induced by supraphysiological doses of combinations of anabolic steroids correlate to the severity of abuse. European Psychiatry, 21(8), 551–562. 10.1016/j.eurpsy.2005.09.001 16356691

[brb33040-bib-0046] Parkinson, A. B. , & Evans, N. A. (2006). Anabolic androgenic steroids: A survey of 500 users. Medicine and Science in Sports and Exercise, 38(4), 644–651. 10.1249/01.mss.0000210194.56834.5d 16679978

[brb33040-bib-0047] Patel, M. S. , Nackeeran, S. , Nallakumar, D. , & Ramasamy, R. (2021). Anabolic‐androgenic steroids are associated with major depressive disorder and suicide attempt: Analysis of a multi‐national database. Fertility and Sterility, 116(3), E348–E348.

[brb33040-bib-0048] Perry, P. J. , Andersen, K. H. , & Yates, W. R. (1990). Illicit anabolic steroid use in athletes. A case series analysis. American Journal of Sports Medicine, 18(4), 422–428. http://www.ncbi.nlm.nih.gov/pubmed/2403192 2403192 10.1177/036354659001800416

[brb33040-bib-0049] Perry, P. J. , Kutscher, E. C. , Lund, B. C. , Yates, W. R. , Holman, T. L. , & Demers, L. (2003). Measures of aggression and mood changes in male weightlifters with and without androgenic anabolic steroid use [Comparative Study]. Journal of Forensic Science, 48(3), 646–651. http://www.ncbi.nlm.nih.gov/pubmed/12762541 12762541

[brb33040-bib-0050] Petersson, A. , Garle, M. , Holmgren, P. , Druid, H. , Krantz, P. , & Thiblin, I. (2006). Toxicological findings and manner of death in autopsied users of anabolic androgenic steroids. Drug and Alcohol Dependence, 81(3), 241–249. 10.1016/j.drugalcdep.2005.07.003 16137840

[brb33040-bib-0051] Piacentino, D. , Sani, G. , Kotzalidis, G. D. , Cappelletti, S. , Longo, L. , Rizzato, S. , Fabi, F. , Frati, P. , Fineschi, V. , & Leggio, L. (2022). Anabolic androgenic steroids used as performance and image enhancing drugs in professional and amateur athletes: Toxicological and psychopathological findings. Human Psychopharmacology, 37(1), e2815. 10.1002/hup.2815 34528289 PMC8727496

[brb33040-bib-0052] Pope, H. G. , Kanayama, G. , Athey, A. , Ryan, E. , Hudson, J. I. , & Baggish, A. (2014). The lifetime prevalence of anabolic‐androgenic steroid use and dependence in Americans: Current best estimates [Research Support, N.I.H., Extramural]. American Academy of Psychiatrists in Alcoholism and Addictions, 23(4), 371–377. 10.1111/j.1521-0391.2013.12118.x PMC3961570 24112239

[brb33040-bib-0053] Pope, H. G. , Kanayama, G. , & Hudson, J. I. (2012). Risk factors for illicit anabolic‐androgenic steroid use in male weightlifters: A cross‐sectional cohort study. Biological Psychiatry, 71(3), 254–261. 10.1016/j.biopsych.2011.06.024 21839424 PMC3218214

[brb33040-bib-0054] Pope, H. G. , Kanayama, G. , Hudson, J. I. , & Kaufman, M. J. (2021). Review article: Anabolic‐androgenic steroids, violence, and crime: Two cases and literature review. American Academy of Psychiatrists in Alcoholism and Addictions, 30(5), 423–432. 10.1111/ajad.13157 PMC8995103 33870584

[brb33040-bib-0055] Pope, H. G. Jr. , & Katz, D. L. (1988). Affective and psychotic symptoms associated with anabolic steroid use. American Journal of Psychiatry, 145(4), 487–490. 10.1176/ajp.145.4.487 3279830

[brb33040-bib-0056] Pope, H. G. , & Katz, D. L. (1994). Psychiatric and medical effects of anabolic‐androgenic steroid use. A controlled study of 160 athletes. Archives of General Psychiatry, 51(5), 375–382. http://www.ncbi.nlm.nih.gov/pubmed/8179461 8179461 10.1001/archpsyc.1994.03950050035004

[brb33040-bib-0057] Pope, H. G. , Wood, R. I. , Rogol, A. , Nyberg, F. , Bowers, L. , & Bhasin, S. (2014). Adverse health consequences of performance‐enhancing drugs: An Endocrine Society scientific statement [Review]. Endocrine Reviews, 35(3), 341–375. 10.1210/er.2013-1058 24423981 PMC4026349

[brb33040-bib-0058] R Core Team . (2018). R: A language and environment for statistical computing. R Foundation for Statistical Computing. https://www.R‐project.org/

[brb33040-bib-0059] Rasmussen, J. J. , Selmer, C. , Østergren, P. B. , Pedersen, K. B. , Schou, M. , Gustafsson, F. , Faber, J. , Juul, A. , & Kistorp, C. (2016). Former abusers of anabolic androgenic steroids exhibit decreased testosterone levels and hypogonadal symptoms years after cessation: A case‐control study. PLoS ONE, 11(8), e0161208. 10.1371/journal.pone.0161208 27532478 PMC4988681

[brb33040-bib-0060] Rosenström, T. , Gjerde, L. C. , Krueger, R. F. , Aggen, S H. , Czajkowski, N. O. , Gillespie, N. A. , Kendler, K. S. , Reichborn‐Kjennerud, T. , Torvik, F. A. , & Ystrom, E. (2019). Joint factorial structure of psychopathology and personality. Psychological Medicine, 49(13), 2158–2167. 10.1017/S0033291718002982 30392478

[brb33040-bib-0061] Sagoe, D. , Mcveigh, J. , Bjørnebekk, A. , Essilfie, M. S. , Andreassen, C. S. , & Pallesen, S. (2015). Polypharmacy among anabolic‐androgenic steroid users: a descriptive metasynthesis. Substance Abuse Treatment, Prevention, and Policy, 10, 12. 10.1186/s13011-015-0006-5 25888931 PMC4377045

[brb33040-bib-0062] Sagoe, D. , Molde, H. , Andreassen, C. S. , Torsheim, T. , & Pallesen, S. (2014). The global epidemiology of anabolic‐androgenic steroid use: a meta‐analysis and meta‐regression analysis. Annals of Epidemiology, 24(5), 383–398. 10.1016/j.annepidem.2014.01.009 24582699

[brb33040-bib-0063] Scarth, M. , & Bjørnebekk, A. (2021). Androgen abuse and the brain. Current Opinion In Endocrinology, Diabetes, and Obesity, 28(6), 604–614. 10.1097/MED.0000000000000675 34709215 PMC8631164

[brb33040-bib-0064] Scarth, M. , Havnes, I. A. , Jørstad, M. L. , Mcveigh, J. , Van Hout, M. C. , Westlye, L. T. , Torgersen, S. , & Bjørnebekk, A. (2022). Severity of anabolic steroid dependence, executive function, and personality traits in substance use disorder patients in Norway. Drug and Alcohol Dependence, 231, 109275. 10.1016/j.drugalcdep.2022.109275 35030506

[brb33040-bib-0065] Scarth, M. , Jørstad, M. L. , Reierstad, A. , Klonteig, S. , Torgersen, S. , & Bjørnebekk, A. (2022). Psychopathology among anabolic‐androgenic steroid users and non‐using female athletes in Norway. Journal of Psychiatric Research, 155, 295–301. 10.31234/osf.io/xrwgs 36170757

[brb33040-bib-0066] Skarberg, K. , Nyberg, F. , & Engstrom, I. (2009). Multisubstance use as a feature of addiction to anabolic‐androgenic steroids. European Addiction Research, 15(2), 99–106. 10.1159/000199045 19182484

[brb33040-bib-0067] Sourander, A. , Jensen, P. , Davies, M. , Niemelä, S. , Elonheimo, H. , Ristkari, T. , Helenius, H. , Sillanmäki, L. , Piha, J. , Kumpulainen, K. , Tamminen, T. , Moilanen, I. , & Almqvist, F. (2007). Who is at greatest risk of adverse long‐term outcomes? The Finnish From a Boy to a Man study. Journal of the American Academy of Child and Adolescent Psychiatry, 46(9), 1148–1161. 10.1097/chi.0b013e31809861e9 17712238

[brb33040-bib-0068] Thiblin, I. , Garmo, H. , Garle, M. , Holmberg, L. , Byberg, L. , Michaëlsson, K. , & Gedeborg, R. (2015). Anabolic steroids and cardiovascular risk: A national population‐based cohort study. Drug and Alcohol Dependence, 152, 87–92. 10.1016/j.drugalcdep.2015.04.013 26005042

[brb33040-bib-0069] Thiblin, I. , Runeson, B. O. , & Rajs, J. (1999). Anabolic androgenic steroids and suicide [Case Reports]. Annals of Clinical Psychiatry, 11(4), 223–231. http://www.ncbi.nlm.nih.gov/pubmed/10596737 10596737 10.1023/a:1022313529794

[brb33040-bib-0070] Tricker, R. , Casaburi, R. , Storer, T. W. , Clevenger, B. , Berman, N. , Shirazi, A. , & Bhasin, S. (1996). The effects of supraphysiological doses of testosterone on angry behavior in healthy eugonadal men–a clinical research center study. Journal of Clinical Endocrinology and Metabolism, 81(10), 3754–3758. 10.1210/jcem.81.10.8855834 8855834

[brb33040-bib-0071] Verheul, R. (2001). Co‐morbidity of personality disorders in individuals with substance use disorders. European Psychiatry, 16(5), 274–282. 10.1016/s0924-9338(01)00578-8 11514129

[brb33040-bib-0072] Westlye, L. T. , Kaufmann, T. , Alnæs, D. , Hullstein, I. R. , & Bjørnebekk, A. (2017). Brain connectivity aberrations in anabolic‐androgenic steroid users. Neuroimage Clinical Applications, 13, 62–69. 10.1016/j.nicl.2016.11.014 PMC5133655 27942448

[brb33040-bib-0073] Windfeld‐Mathiasen, J. , Christoffersen, T. , Strand, N. A. W. , Dalhoff, K. , Andersen, J. T. , & Horwitz, H. (2022). Psychiatric morbidity among men using anabolic steroids. Depression and Anxiety, 39(12), 805–812. 10.1002/da.23287 36281632 PMC10092709

[brb33040-bib-0074] Yates, W. R. , Perry, P. J. , & Andersen, K. H. (1990). Illicit anabolic steroid use: A controlled personality study. Acta Psychiatrica Scandinavica, 81(6), 548–550. http://www.ncbi.nlm.nih.gov/pubmed/2378247 2378247 10.1111/j.1600-0447.1990.tb05496.x

