# Supplementary material for: Clustering psychopathology in male anabolic–androgenic steroid users and nonusing weightlifters
Source: Brain Behav. 2023 May 7;13(7):e3040. doi: 10.1002/brb3.3040 (PMC10338822; doi:10.1002/brb3.3040)
Supplement: Supplementary file 1 — Supplementary Figure 1: NbClust showing the optimal number of clusters. Supplementary Figure 2: Elbow plot showing the optimal number of clusters. Supplementary Figure 3. Dendrogram showing how the four clusters divide from each other. Supplementary Table 1. Frequency and percent of MCMI‐III scales above the clinical cut‐off (≥75). Supplementary Table 2: Characteristics of MCMI‐III psychopathology sub‐groups. Supplementary Table 3: Current and previous AAS users, MCMI‐III base rate medians, interquartile range and results of the Wilcoxon‐Mann‐Whitney tests. Supplementary Table 4: Current and previous AAS users, frequency and percent of MCMI‐III scales above the clinical cut‐off (≥75). [file BRB3-13-e3040-s001.docx]

**Appendix**

**Supplementary Table 1.** Frequency and percent of MCMI-III scales above the clinical cut-off (≥75).

|  | **Non-Using Weightlifters (n=97)** | | **AAS**  **Users**  **(n=117)** | | ***p*** |
| --- | --- | --- | --- | --- | --- |
|  | **BR ≥75** | | **BR ≥75** | |  |
| **MCMI-III scales** | **n** | **%** | **n** | **%** |  |
| Moderate Personality Disorder Scales |  |  |  |  |  |
| Schizoid | 7 | 7.2 | 22 | 18.8 | 0.03 |
| Avoidant | 5 | 5.2 | 24 | 20.5 | 0.00 |
| Depressive | 7 | 7.2 | 37 | 31.6 | 0.00 |
| Dependent | 6 | 6.2 | 23 | 19.7 | 0.01 |
| Histrionic | 0 | 0 | 0 | 0 | NA |
| Narcissistic | 7 | 7.2 | 13 | 11.1 | 0.43 |
| Antisocial | 3 | 3.1 | 27 | 23.1 | 0.00 |
| Aggressive (Sadistic) | 1 | 1.0 | 8 | 6.8 | 0.06 |
| Compulsive | 0 | 0 | 0 | 0 | NA |
| Passive Aggressive (Negativistic) | 2 | 2.1 | 22 | 18.8 | 0.00 |
| Self-Defeating (Masochistic) | 4 | 4.1 | 28 | 23.9 | 0.00 |
| Severe Personality Pathology Scales |  |  |  |  |  |
| Schizotypal | 0 | 0.0 | 8 | 6.8 | 0.02 |
| Borderline | 1 | 1.0 | 15 | 12.8 | 0.00 |
| Paranoid | 1 | 1.0 | 3 | 2.6 | 0.72 |
| Moderate Clinical Syndrome Scales |  |  |  |  |  |
| Anxiety | 9 | 9.3 | 39 | 33.3 | 0.00 |
| Somatoform | 0 | 0.0 | 4 | 3.4 | 0.16 |
| Bipolar | 0 | 0.0 | 7 | 6.0 | 0.03 |
| Dysthymia | 2 | 2.1 | 24 | 20.5 | 0.00 |
| Alcohol | 1 | 1.0 | 19 | 16.2 | 0.00 |
| Drug | 1 | 1.0 | 18 | 15.4 | 0.00 |
| Post-Traumatic Stress Disorder | 0 | 0.0 | 9 | 7.7 | 0.01 |
| Severe Syndrome Scales |  |  |  |  |  |
| Thought disorder | 0 | 0.0 | 5 | 4.3 | 0.09 |
| Major depression | 0 | 0.0 | 6 | 5.1 | 0.05 |
| Delusional | 0 | 0.0 | 1 | 0.9 | 1.00 |

Fisher's exact test was used for comparing the groups on the dichotomized MCMI-III scales (number of individuals above the clinical cut-off on BR ≥75). P-values were adjusted using Benjamini-Hochberg procedure. AAS, anabolic androgenic steroids

**
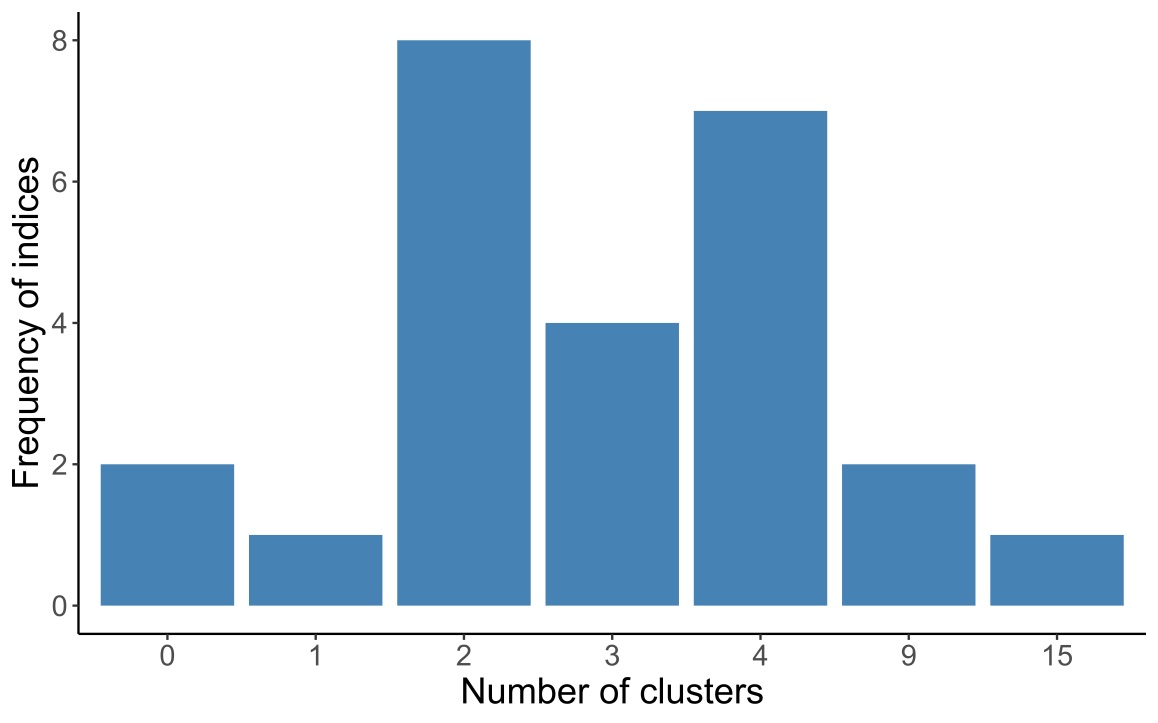
**

**Supplementary Figure 1:** NbClust showing the optimal number of clusters.

**
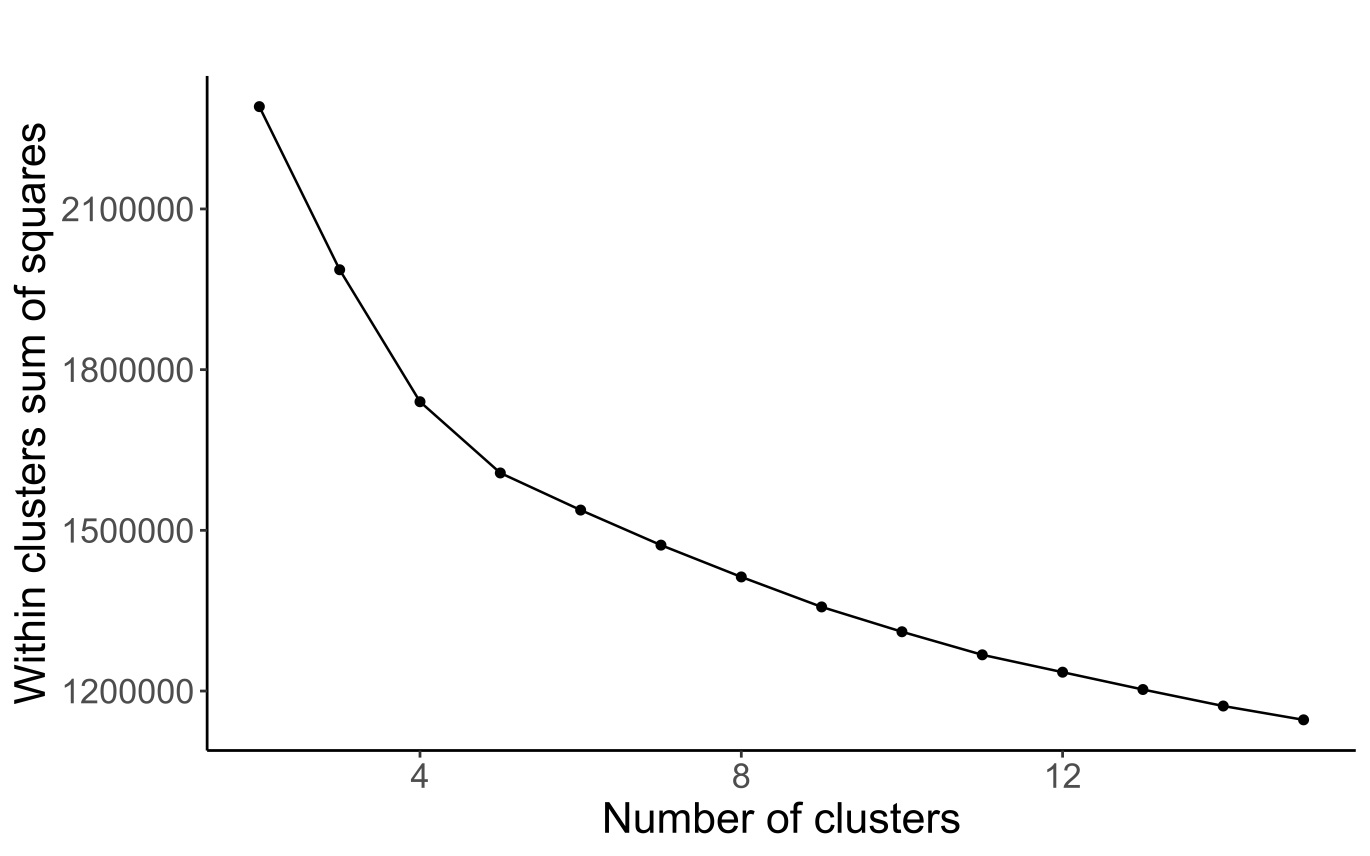
**

**Supplementary Figure 2:** Elbow plot showing the optimal number of clusters.


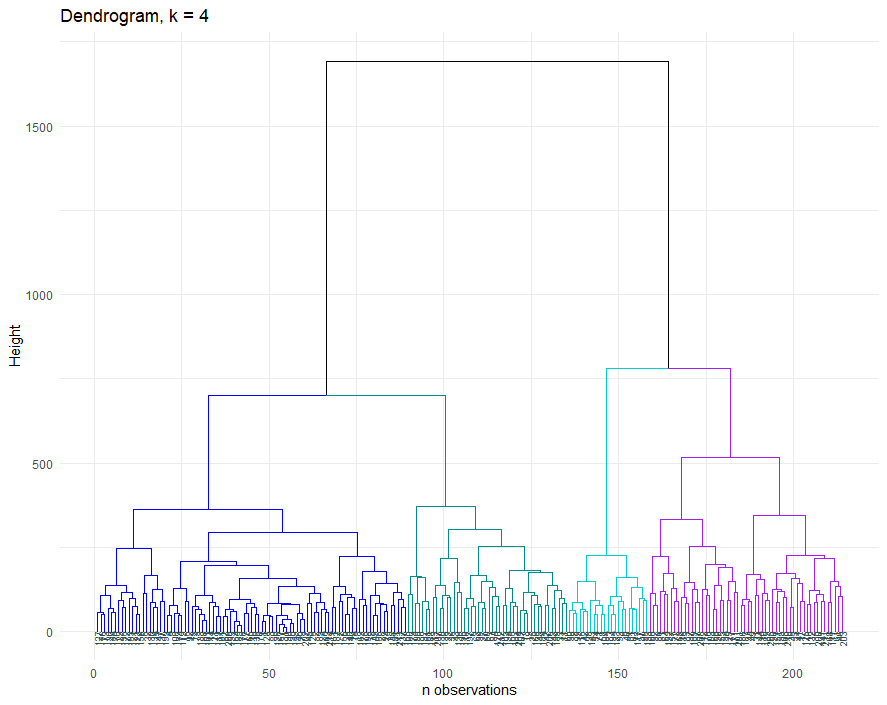


**Supplementary Figure 3.** Dendrogram showing how the four clusters divide from each other.

**Supplementary Table 2:** Characteristics of MCMI-III psychopathology sub-groups.

|  | **Cluster 1 (n=89)**  «No Psychopathology» | **Cluster 2 (n=46)**  «Mild Externalizing» | **Cluster 3 (n=23)**  «Severe Multi-Pathology» | **Cluster 4 (n=56)**  «Mild Multi-Pathology» |  |
| --- | --- | --- | --- | --- | --- |
|  | **Mean (SD)** | **Mean (SD)** | **Mean (SD)** | **Mean (SD)** | ***p*** |
| Age | 36.29 (10.26) | 37.85 (10.64) | 33.26 (8.38) | 34.59 (9.68) | 0.222 |
| Education (years) | 16.60 (2.61)^2, 3, 4^ | 14.70 (2.66)^1^ | 14.33 (2.46)^1^ | 14.63 (3.11)^1^ | <0.001 |
| Age at onset of AAS use | 24.23 (6.33)^3^ | 24.06 (8.46)^3^ | 18.18 (3.32)^1,2^ | 22.84 (8.60) | 0.03 |
| Total years used | 10.38 (8.49) | 10.53 (7.95) | 11.84 (9.04) | 10.24 (7.16) | 0.909 |
| Weekly dose (n(%)) |  |  |  |  | 0.28 |
| Low | 5 (5.6) | 2 (4.3) | 2 (8.7) | 2 (3.6) |  |
| Medium | 14 (15.7) | 12 (26.1) | 7 (30.4) | 19 (33.9) |  |
| High | 5 (5.6) | 14 (30.4) | 7 (30.4) | 11 (19.6) |  |
| NA | 2 (2.2) | 5 (10.9) | 5 (21.7) | 5 (8.9) |  |

**Supplementary Table 3:** Current and previous AAS users, MCMI-III base rate medians, interquartile range and results of the Wilcoxon-Mann-Whitney tests.

|  | **Current AAS users**  **(n=72)** | | **Previous AAS users**  **(n=32)** | | **Z** | ***p*** |
| --- | --- | --- | --- | --- | --- | --- |
| **MCMI-III scales** | **Median** | **IQR** | **Median** | **IQR** |  |  |
| Moderate Personality Disorder Scales |  |  |  |  |  |  |
| Schizoid | 48.00 | [24.00, 68.00] | 68.00 | [36.00, 75.00] | -2,41 | 0.35 |
| Avoidant | 24.00 | [12.00, 63.50] | 48.00 | [24.00, 78.25] | -1,96 | 0.35 |
| Depressive | 40.00 | [0.00, 75.00] | 62.00 | [15.00, 85.00] | -1,36 | 0.52 |
| Dependent | 30.00 | [20.00, 70.00] | 50.00 | [30.00, 71.25] | -1,04 | 0.54 |
| Histrionic | 40.00 | [34.00, 44.00] | 39.00 | [26.00, 44.50] | 0,23 | 0.82 |
| Narcissistic | 59.00 | [51.00, 63.50] | 57.00 | [53.00, 61.00] | 0,46 | 0.71 |
| Antisocial | 60.00 | [30.00, 73.00] | 56.00 | [38.00, 80.00] | -0,97 | 0.54 |
| Aggressive (Sadistic) | 60.00 | [26.00, 62.00] | 61.00 | [26.00, 63.25] | -0,9 | 0.54 |
| Compulsive | 44.00 | [35.50, 49.00] | 44.00 | [36.00, 47.50] | 0,24 | 0.82 |
| Passive Aggressive (Negativistic) | 22.00 | [15.00, 60.00] | 38.00 | [15.00, 80.00] | -1,01 | 0.54 |
| Self-Defeating (Masochistic) | 20.00 | [0.00, 68.00] | 60.00 | [0.00, 75.25] | -1,92 | 0.35 |
| Severe Personality Pathology Scales |  |  |  |  |  |  |
| Schizotypal | 40.00 | [0.00, 63.00] | 40.00 | [35.00, 67.25] | -1,64 | 0.43 |
| Borderline | 30.00 | [10.00, 60.00] | 40.00 | [20.00, 62.75] | -0,87 | 0.54 |
| Paranoid | 48.00 | [12.00, 63.00] | 62.50 | [24.00, 68.50] | -1,89 | 0.35 |
| Moderate Clinical Syndrome Scales |  |  |  |  |  |  |
| Anxiety | 20.00 | [0.00, 78.50] | 40.00 | [0.00, 85.00] | -0,45 | 0.71 |
| Somatoform | 60.00 | [0.00, 63.00] | 60.00 | [0.00, 68.00] | -0,7 | 0.61 |
| Bipolar: Manic | 60.00 | [24.00, 62.00] | 60.00 | [36.00, 66.50] | -0,94 | 0.54 |
| Dysthymia | 20.00 | [0.00, 61.00] | 30.00 | [0.00, 79.25] | -1,61 | 0.43 |
| Alcohol Dependence | 45.00 | [41.25, 65.00] | 37.50 | [26.25, 70.00] | 0,46 | 0.71 |
| Drug Dependence | 60.00 | [30.00, 68.00] | 62.50 | [41.25, 69.00] | -0,8 | 0.57 |
| Post-Traumatic Stress Disorder | 15.00 | [0.00, 63.00] | 30.00 | [0.00, 68.00] | -1,91 | 0.54 |
| Severe Syndrome Scales |  |  |  |  |  |  |
| Thought Disorder | 22.50 | [0.00, 61.50] | 45.00 | [15.00, 67.00] | -1,53 | 0.43 |
| Major Depression | 20.00 | [0.00, 60.25] | 40.00 | [0.00, 65.00] | -1,28 | 0.53 |
| Delusional Disorder | 25.00 | [0.00, 60.75] | 42.50 | [18.75, 63.00] | -1,1 | 0.54 |

AAS, anabolic androgenic steroids; IQR, interquartile range

P-values were adjusted using Benjamini-Hochberg procedure.

**Supplementary Table 4:** Current and previous AAS users, frequency and percent of MCMI-III scales above the clinical cut-off (≥75).

|  | **Current AAS users**  **(n=72)** | | **Previous AAS users**  **(n=32)** | | ***p*** |
| --- | --- | --- | --- | --- | --- |
|  | **BR ≥75** | | **BR ≥75** | |  |
| **MCMI-III scales** | **n** | **%** | **n** | **%** |  |
| Moderate Personality Disorder Scales |  |  |  |  |  |
| Schizoid | 9 | 12.5 | 11 | 34.4 | 0.34 |
| Avoidant | 12 | 16.7 | 10 | 31.2 | 0.41 |
| Depressive | 20 | 27.8 | 14 | 43.8 | 0.41 |
| Dependent | 15 | 20.8 | 8 | 25.0 | 0.87 |
| Histrionic | 0 |  | 0 |  | NA |
| Narcissistic | 9 | 12.5 | 3 | 9.4 | 0.93 |
| Antisocial | 15 | 20.8 | 10 | 31.2 | 0.59 |
| Aggressive (Sadistic) | 2 | 2.8 | 4 | 12.5 | 0.41 |
| Compulsive | 0 |  | 0 |  | NA |
| Passive Aggressive (Negativistic) | 10 | 13.9 | 9 | 28.1 | 0.41 |
| Self-Defeating (Masochistic) | 17 | 23.6 | 10 | 31.2 | 0.71 |
| Severe Personality Pathology Scales |  |  |  |  |  |
| Schizotypal | 4 | 5.6 | 3 | 9.4 | 0.90 |
| Borderline | 7 | 9.7 | 6 | 18.8 | 0.59 |
| Paranoid | 1 | 1.4 | 2 | 6.2 | 0.59 |
| Moderate Clinical Syndrome Scales |  |  |  |  |  |
| Anxiety | 23 | 31.9 | 14 | 43.8 | 0.59 |
| Somatoform | 1 | 1.4 | 3 | 9.4 | 0.41 |
| Bipolar | 5 | 6.9 | 0 | 0.0 | 0.59 |
| Dysthymia | 11 | 15.3 | 11 | 34.4 | 0.41 |
| Alcohol | 10 | 13.9 | 7 | 21.9 | 0.62 |
| Drug | 11 | 15.3 | 6 | 18.8 | 0.93 |
| Post-Traumatic Stress Disorder | 4 | 5.6 | 4 | 12.5 | 0.59 |
| Severe Syndrome Scales |  |  |  |  |  |
| Thought disorder | 4 | 5.6 | 1 | 3.1 | 1.00 |
| Major depression | 3 | 4.2 | 3 | 9.4 | 0.62 |
| Delusional | 1 | 1.4 | 0 | 0.0 | 1.00 |

AAS, anabolic androgenic steroids.
P-values were adjusted using Benjamini-Hochberg procedure.
